# Hypophosphataemia after ferric carboxymaltose is unrelated to symptoms, intestinal inflammation or vitamin D status

**DOI:** 10.1186/s12876-020-01298-9

**Published:** 2020-06-10

**Authors:** Wendy Fang, Rachel Kenny, Qurat-ul-Ain Rizvi, Lawrence P. McMahon, Mayur Garg

**Affiliations:** 1 Department of Gastroenterology, Northern Health, 185 Cooper St, Epping, Victoria 3076 Australia; 2 Eastern Health Clinical School, Monash University, 5 Arnold St, Box Hill, Victoria 3128 Australia; 3 Department of Nephrology, Eastern Health, 8 Arnold St, Box Hill, Victoria 3128 Australia; 4 Department of Renal Medicine, Box Hill Hospital, Level 3W, Building B, 8 Arnold St, Box Hill, Victoria 3128 Australia; 5 Department of Gastroenterology, Northern Health, Epping, Victoria Australia; 6 Department of Gastroenterology and Hepatology, Royal Melbourne Hospital, Parkville, Victoria Australia; 7 Department of Gastroenterology and Hepatology, Royal Melbourne Hospital, Parkville, Victoria Australia

**Keywords:** Iron deficiency, Ferric carboxymaltose, Inflammatory bowel disease, Hypophosphataemia, Vitamin D

## Abstract

**Background:**

Intravenous iron replacement is recommended for iron-deficient patients with inflammatory bowel disease (IBD), but may be associated with hypophosphataemia, predisposing to osteomalacia and fractures. This study aimed to evaluate the incidence and risk factors for hypophosphataemia following intravenous ferric carboxymaltose (FCM) in patients with IBD.

**Methods:**

This prospective observational study of patients with and without IBD evaluated serum phosphate for 28 days following intravenous FCM, and assessed associations with symptoms, markers of inflammation and vitamin D status.

**Results:**

Twenty-four patients with IBD (11 with Crohn’s disease [CD], 13 with ulcerative colitis [UC], mean age 45 years [range 19–90], 7 female), and 20 patients without IBD (mean age 56 [22–88] y, 11 female), were included. Overall, serum phosphate declined by a mean of 36% at Day 7, with a mean fall of 42% (SD 19%) at some time point over 28 days (*p* <  0.001). Twenty-four of 44 (55%) patients developed moderate to severe hypophosphataemia (serum phosphate < 0.6 mmol/L). No differences between patients with and without IBD were seen, but patients with CD had greater decline in phosphate than those with UC. There was no association between hypophosphataemia and symptomatic adverse events, faecal calprotectin, C-reactive protein, albumin, platelet count, 25(OH) vitamin D, or 1,25(di-OH) vitamin D. Serum phosphate < 1.05 mmol/L on Day 2 predicted susceptibility to moderate-severe hypophosphataemia (OR 7.0).

**Conclusions:**

Hypophosphataemia following FCM is common, unrelated to symptomatic adverse events, baseline intestinal or systemic inflammation, or vitamin D status.

## Background

Iron deficiency, with or without anaemia, is one of the commonest systemic complications in patients with inflammatory bowel disease (IBD), affecting between 13 and 90% of patients [1–4]. Iron deficiency anaemia in patients with IBD is considered a marker of disease activity, and is associated with a reduced quality of life, impaired cognition and social functioning, and an increased risk of hospitalisation [5–7]. Recognition and correction of iron deficiency independent of IBD activity is associated with an improvement in these parameters [7, 8], and intravenous iron is recommended for patients with moderate to severe anaemia or intolerance to oral iron formulations [2, 4, 5, 9].

Modern intravenous iron formulations are considered safe in patients with IBD, with treatment-related adverse events described in 10–30% of patients, including nasopharyngitis, headaches, back pain, nausea or flu-like symptoms [10–14].

Hypophosphataemia following intravenous iron, especially following ferric carboxymaltose (FCM), is increasingly recognised, likely mediated by a transient increase in intact fibroblast growth factor-23 (iFGF-23) due to inhibition of its cleavage, which results in phosphaturia [15–17]. When recurrent or persistent, this may result in osteomalacia and fractures [16, 18].

Of particular relevance to patients with IBD, repeated iron infusions, malnutrition and vitamin D deficiency may aggravate risk of hypophosphataemia [19]. Furthermore, iFGF-23 is upregulated by systemic inflammation, which may, theoretically, be a further risk factor for hypophosphataemia in patients with IBD [20]. This study aimed to prospectively evaluate the incidence and severity of intravenous FCM-associated hypophosphataemia in patients with and without IBD, its association with symptoms, and ascertain associated risk factors including systemic and intestinal inflammation, and vitamin D status, to enable potential preventative strategies.

## Methods

### Subjects

Consecutive non-pregnant iron-deficient patients with and without IBD attending a tertiary gastroenterology unit, who were deemed to require intravenous FCM as per clinician judgement, were invited to participate. Patients had evidence of iron deficiency based upon ferritin < 30 ng/ml or ferritin < 100 ng/ml with evidence of blood loss or inflammation. Non-IBD controls had iron deficiency secondary to occult or overt gastrointestinal bleeding (*n* = 16) or menstrual blood loss (*n* = 4).

### Protocol and analytical assays

One gram FCM was administered to all patients. Baseline demographics and disease characteristics, haematological and biochemical indices, 25-hydroxy vitamin D and serum for analysis of iFGF-23, c-terminal FGF-23 (cFGF-23, the cleaved fragment of iFGF-23) and vitamin D binding protein (DBP), were collected. Faecal samples were analysed for calprotectin by fluorescence enzyme immunoassay (Phadia 100 EliA™ Calprotectin, Thermo Scientific, Scoresby, Australia). Clinical and biochemical assessment was repeated 2, 4, 7, 14 and 28 days after infusion, including direct questioning of gastrointestinal symptoms and adverse events, and serum stored for analysis of iFGF-23 and cFGF-23 at Days 2, 7 and 28.

Serum iFGF-23 was analysed in duplicate by enzyme-linked immunosorbent assay (ELISA, Kainos, Shizuoka, Japan), as previously described [21]. Serum cFGF-23 was analysed in duplicate by ELISA (Immunotopics, San Clemente, CA, USA), as described previously [21].

### Endpoints

The endpoints for this study included mean reduction in serum phosphate levels from Day 0 to Day 7, the proportion of patients experiencing a moderate (Grade 3, serum phosphate < 0.6 mmol/L) to severe (Grade 4 toxicity, < 0.3 mol/L) hypophosphataemia according to Common Terminology Criteria for Adverse Events (CTCAE) [22] at any stage during the follow-up period, difference in rate of hypophosphataemia between patients with and without IBD, and correlation between hypophosphataemia and symptomatic adverse events, also graded according to CTCAE. The association of hypophosphataemia with degree of systemic (C-reactive protein) or intestinal (faecal calprotectin) inflammation, and serum vitamin D status was additionally evaluated.

### Statistical analyses

Results were analysed by SPSS v23 (IBM Corp) and Graphpad Prism v6 (GraphPad Software, Inc., California, USA) using paired and unpaired t-tests, Fisher’s exact test, and multiple regression analyses as appropriate. A two-tailed *p*-value of < 0.05 was considered statistically significant for all associations.

### Ethical considerations

The protocol for this study was approved by the Office of Research and Ethics at Eastern Health (LR 17–2017, approved 17 March 2017), and was performed in accordance with Australian regulations and the principles of the Declaration of Helsinki 1954 and its later amendments. Written, informed consent was obtained from all participants included in this study.

## Results

Twenty-three non-IBD controls and 27 patients with IBD were recruited. Three patients from each group were excluded due to loss of follow-up within 1 week following infusion, leaving 24 patients with IBD (11 with Crohn’s disease [CD] and 13 with ulcerative colitis [UC)]) and 20 non-IBD controls for analysis (Table 1). Characteristics of disease in patients with IBD are shown in Table 2.

**Table 1:** Baseline participant characteristics

	**IBD (*n* = 24)**	**Non-IBD Controls (*n* = 20)**	***P*value**^**†**^
**Age (mean +/− range)**	45 (19–90)	56 (22–88)	0.045
**Female:Male**	7:17	11:9	0.153^‡^
**Co-morbid illnesses, n (%)**			
**Type 2 diabetes mellitus**	2	3	
**Chronic kidney disease**	1	0	
**Atrial fibrillation**	1	0	
**Ischaemic heart disease**	1	2	
**Colorectal adenocarcinoma**	0	1	
**Other cancer**	1	3	
**Body mass index (mean +/− range)**	26.9 (17.6–51.3)	26.5 (5.43)	0.866
**Haemoglobin (g/L, mean, range)**	121 (62–153)	124 (86–162)	0.597
**MCV (10**^**−15**^**L, mean, range)**	82 (55–96)	81 (68–88)	0.601
**Serum ferritin (ng/mL, mean, range)**	30 (3–93)	25 (7–62)	0.395
**White cell count (× 10**^**9**^**/L, mean, range)**	7.6 (4.0–10.9)	6.8 (3.7–21.4)	0.416
**Platelet count (× 10**^**9**^**/L, mean, range)**	318 (150–680)	237 (143–366)	**0.009**
**eGFR (mL/min/1.73m**^**2**^**, mean, range)**	85 (38 - > 90)	80 (45- > 90)	0.273
**Serum albumin (g/L, mean, range)**	35 (25–48)	38 (30–50)	0.064
**Serum C-reactive protein (mg/L, median, range)**	3 (< 2–18)	< 2 (< 2–15)	0.228
**Faecal calprotectin (**μg**/g, median, range)**	573 (< 15- > 3000)	47 (< 15–300)	**0.023**
**25(OH) vitamin D (nmol/L, mean, range)**	51 (8–104)	49 (20–96)	0.822
**1,25(di-OH) vitamin D (pmol/L, mean, range)**	112 (50–202)	136 (62–263)	0.109
**Serum calcium (corrected, mmol/L, mean** ± **95% CI)**	2.40 (2.29–2.49)	2.36 (2.21–2.55)	0.115
**Serum phosphate (mmol/L, mean** ± **95% CI)**	1.11 (0.69–1.39)	1.09 (0.63–1.60)	0.700
**Serum parathyroid hormone (pmol/L, mean** ± **95% CI)**	5.1 (1.9–12.1)	6.3 (1.4–11.5)	0.103

^†^Unpaired t-test unless otherwise specified

^‡^Chi-square

**Table 2:** Characteristics of patients with IBD

	**Crohn’s disease (*n* = 11)**		**Ulcerative colitis (*n* = 13)**	
**Montreal Classification** Number of patients, n	**Age at diagnosis (y)**		**Disease extent**	
	< 17	2	Proctitis	0
	17–40	6	Left sided colitis	7
	> 40	3	Extensive colitis	6
	**Location**		**Disease severity**	
	Ileal	7	Clinical remission	0
	Colonic	2	Mild	0
	Ileocolonic	2	Moderate	8
	Upper GI	0	Severe	5
	**Behavior**			
	Non-stricturing, non-penetrating	4		
	Stricturing	5		
	Penetrating / fistulising	2		
	Perianal	1		
**Disease activity indices**	Harvey Bradshaw Index (median, range)	3 (0–8)	Simple Clinical Colitis Activity Index (median, range)	2 (0–9)
**Medical therapy,** Number of patients, n	Nil	0		0
	5-ASAs only	2		1
	Steroids ±5-ASAs	1		3
	Azathioprine / 6-MP ± 5-ASAs / steroids	1		3
	Methotrexate ±5-ASAs / steroids	0		1
	IFX / ADA / vedolizumab ±5-ASAs / steroids	2		2
	IFX / ADA / vedolizumab ± immunomodulators	5		3
**Previous intestinal surgery / surgeries,** Number of patients, n (%)	Jejunal resection	0		
	Single ileo-colonic resection	3		0
	Multiple ileo-colonic resections	1		0
	Colectomy (total or subtotal)	0		1

*5ASAs* 5-aminosalicylates, *6-MP* 6-mercaptopurine, *IFX* Infliximab, *ADA* Adalimumab

Mean plasma haemoglobin improved similarly (*p* = 0.460) after 28 days in patients with (121 to 134 g/L) and without IBD (124 to 132 g/L).

### Change in serum phosphate

**Fig. 1:**
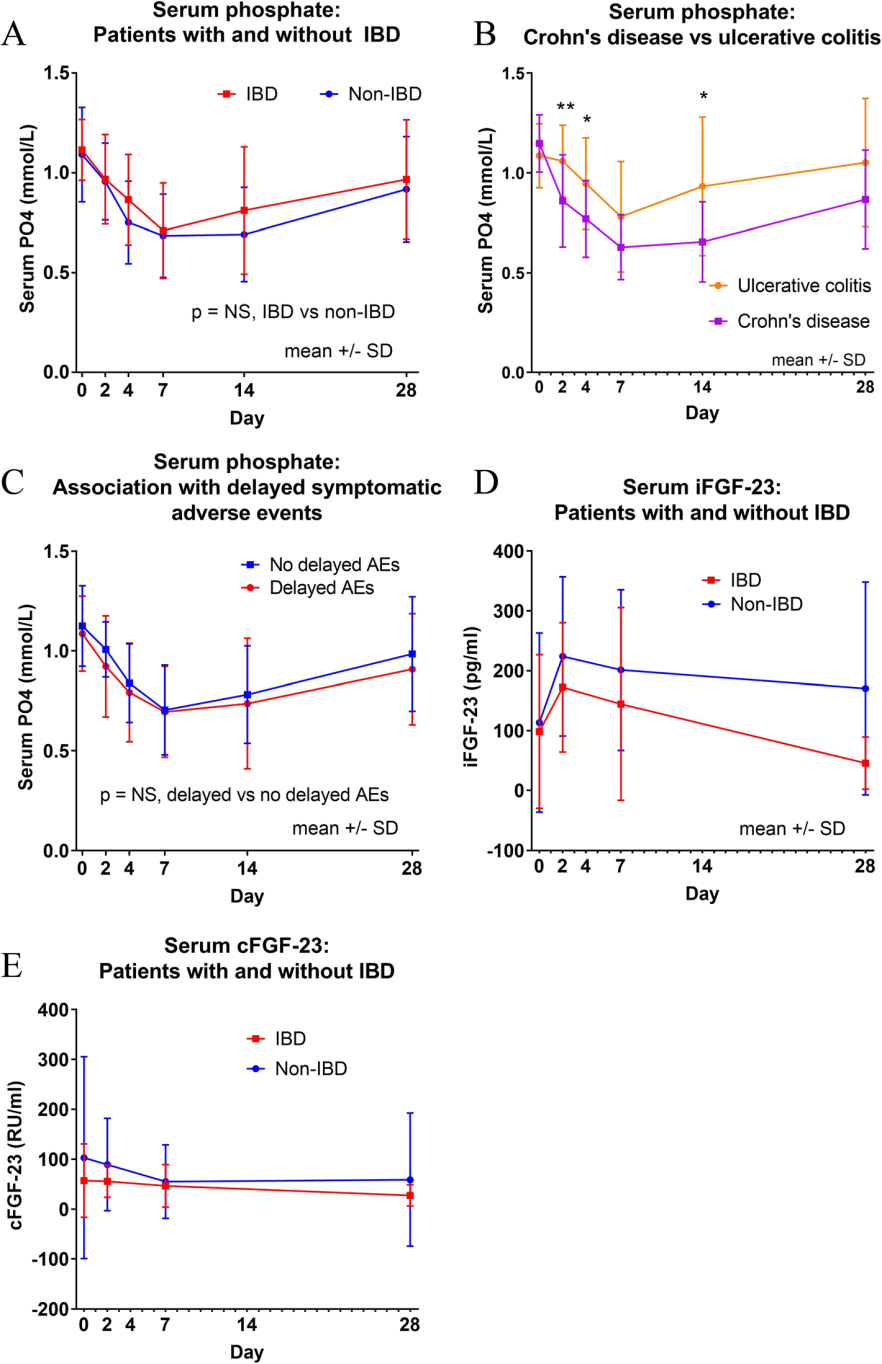
Change in (**a**) serum phosphate following ferric carboxymaltose infusion in patients with and without IBD; **b** change in serum phosphate in patients with Crohn’s disease and ulcerative colitis; **c** association with symptomatic adverse events; **d** change in serum iFGF-23 and (**e**) cFGF-23 over the study period

Serum phosphate fell in 42 of 44 patients (95%) following FCM. The overall mean fall in serum phosphate across all patients and time periods was 0.47 mmol/L ± SD 0.24 (42 ± 19%) compared with baseline. The lowest serum phosphate recorded was 0.26 mmol/L in a patient with UC at Day 7. Mean lowest serum phosphate levels were 36% lower at Day 7, and remained lower at Day 28 compared to baseline (mean 0.95 vs 1.10 mmol/L, *p* = 0.001) (Fig. 1a). Three patients had serum phosphate below 0.6 mmol/L at Day 28 (Table 3). The time to lowest serum phosphate was 2 days in 1 patient, 4 days in 8 patients, 7 days in 22 patients, 14 days in 10 patients, and 28 days in 3 patients.

**Table 3:** Number of patients with moderate to severe hypophosphataemia (serum PO4 < 0.6 mmol/L) according to visit day following ferric carboxymaltose (FCM)

**Visit day**	**IBD (*n* = 24)**	**Non-IBD (*n* = 20)**
**Day 0**	0	0
**Day 2**	1	2
**Day 4**	3	4
**Day 7**	9	5
**Day 14**	7	10
**Day 28**	1	2

Serum phosphate fell below 0.6 mmol/L in 24 of 44 patients (56%), similar in patients with and without IBD (Table 4).

**Table 4:** Number of patients and degree of hypophosphataemia noted in first 28 days following ferric carboxymaltose (FCM)

**Grade of hypophosphataemia (mmol/L)**	**IBD (*n* = 24)**	**Non-IBD (*n* = 20)**
**Nil or Grade 1 (≥ 0.80)**	7	5
**Grade 2 (0.60 - < 0.80)**	4	4
**Grade 3 (0.30 - < 0.60)**	12	11
**Grade 4 (< 0.30)**	1	0

Patients with CD had a significantly greater maximal reduction in serum phosphate than patients with UC (mean reduction 51% vs 32%, *p* = 0.029) (Fig. 1b).

### Symptomatic adverse events

Delayed adverse events were described by 11 patients with IBD (46%) and 12 without IBD (60%) (Supplementary Table 1). Fatigue, arthralgia, myalgia, headache, dyspnoea or dizziness were reported by 14 patients (6 with and 8 without IBD) in the first 7 days – these adverse events did not correlate with serum phosphate (Fig. 1c).

### Change in FGF-23

Baseline iFGF-23 and cFGF-23 levels were similar in patients with and without IBD. Across all patients, iFGF-23 increased by a mean of 84% (95% CI 26–139%, *p* = 0.004), peaking within 2 days, returning to baseline at Day 28. C-terminal FGF-23 levels remained stable at day 2, but gradually declined over time, with a significant reduction at Day 28 compared with baseline levels (*p* = 0.004, paired t-test) (Fig. 1d and e).

### Predictors of hypophosphataemia

**Fig. 2:**
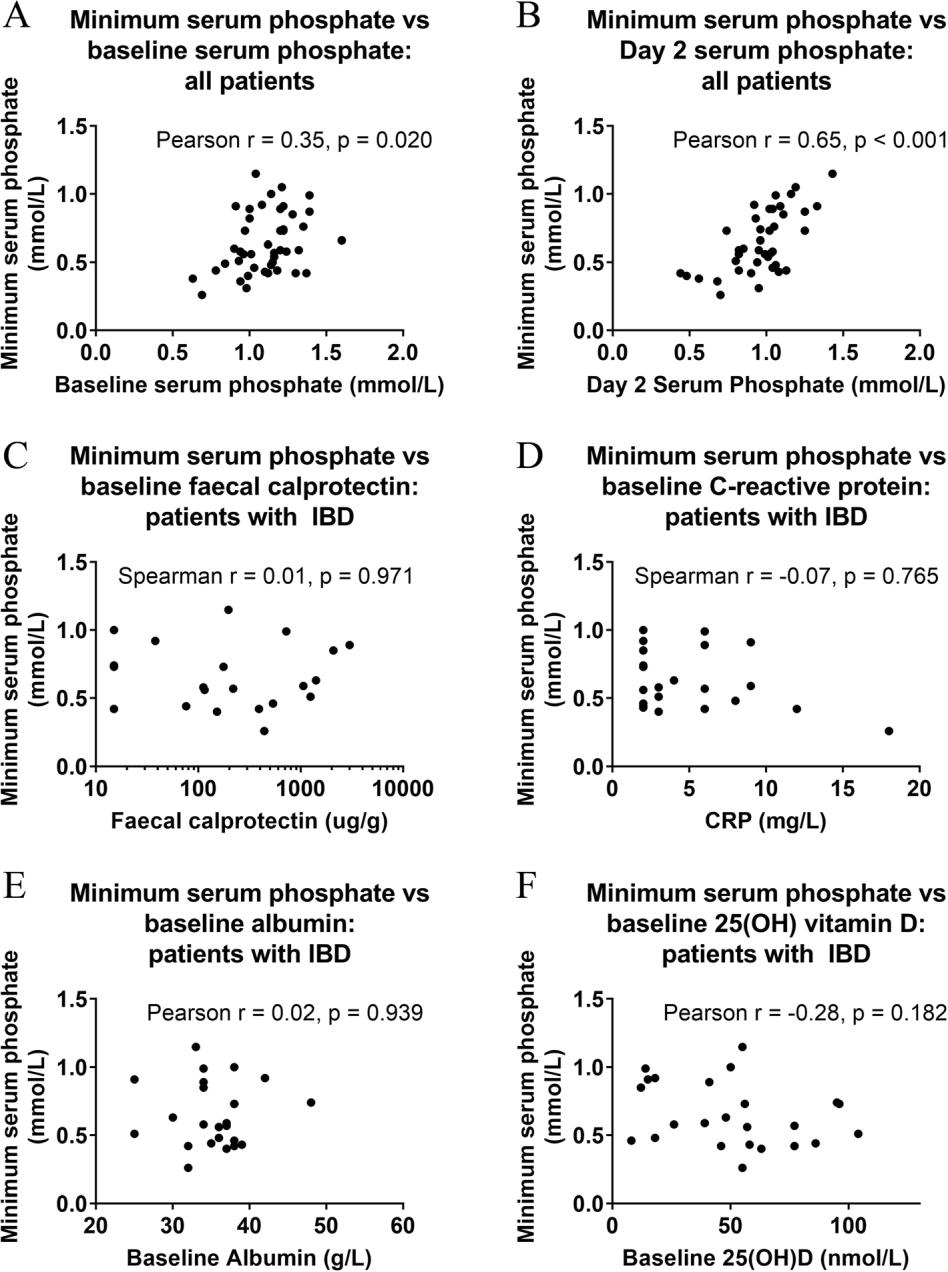
Correlation between minimum serum phosphate during follow-up and (**a**) baseline and (**b**) Day 2 serum phosphate, across all participants; **c** faecal calprotectin, **d** C-reactive protein, **e** albumin and (**f**) 25(OH) vitamin D in patients with IBD

Across all participants, minimum serum phosphate correlated with baseline (Pearson *r* = 0.35, *p* = 0.020) and Day 2 (Pearson *r* = 0.65, *p* <  0.001) phosphate levels (Fig. 2a and b).

In patients with IBD, there was no correlation between minimum serum phosphate and markers of inflammation (faecal calprotectin, C-reactive protein, albumin, platelet count) or baseline 25-hydroxy vitamin D (Fig. 2c-f).

A significant correlation between serum phosphate at Day 2 and minimum phosphate (*r* = 0.67, *p* < 0.001) was noted (Supplementary Figure 1). Minimum serum phosphate did correlate with DBP (*r* = 0.48, *p* = 0.017) but showed a trend towards an inverse correlation with free 25-hydroxy vitamin D (*r* = − 0.39, *p* = 0.060) and less so with bioavailable 25(di-OH) D (*r* = − 0.32, *p* = 0.124) (Supplementary Figure 1).

On multiple regression analysis across all patients, baseline serum phosphate, Day 2 phosphate, C-reactive protein, 25(OH) vitamin D, 1,25(di-OH) vitamin D, were evaluated as independent risk factors for minimum serum phosphate in the follow-up period. The strongest predictor was Day 2 phosphate (β 0.577, *p* < 0.001), with baseline phosphate no longer significant (β 0.165, *p* = 0.259, adjusted R square 0.387). For patients with IBD only, a separate multiple regression model consisting of Day 2 phosphate, faecal calprotectin, DBP and free 25-hydroxy vitamin D was developed. Only Day 2 phosphate (β 0.533, *p* = 0.052) approached significance (adjusted R square 0.344).

When serum phosphate at Day 2 was ≥1.05 mmol/L, the risk of Grade 3 or 4 hypophosphataemia during follow-up was 23% compared to a 67% risk (odds ratio 7.0, 95% CI 1.6–32.0) when Day 2 phosphate was < 1.05 mmol/L.

## Discussion

The risk of hypophosphataemia in patients receiving intravenous FCM is increasingly recognised; however, the risk in patients with IBD compared with patients without IBD, and associated predisposing factors, have not been previously reported. This prospective observational study demonstrated a mean phosphate reduction of 42% following FCM, similar in patients with and without IBD, with more than half the patients experiencing moderate to severe hypophosphataemia. Although patients with CD more frequently experienced hypophosphataemia than patients with UC, patients with IBD per se were not at greater risk than patients without. Importantly, neither the severity of inflammation (assessed by circulating or faecal markers) nor baseline vitamin D status predicted risk of hypophosphataemia.

Most cases of FCM-associated hypophosphataemia are asymptomatic. Indeed, our data show that delayed adverse events secondary to FCM have no relationship with hypophosphataemia, with the 6 patients with IBD and 8 patients without IBD who experienced fatigue, arthralgia, myalgia, headache and dyspnoea or dizziness have similar phosphate levels to those participants without these symptoms. Hence, hypophosphatemia is difficult to recognise unless specifically measured in the serum. In the largest randomised clinical trial of patients with IBD who received FCM, mean serum phosphate was noted to fall by 38% from baseline (1.12 ± 0.22 mmol/L) to Week 2 (0.69 ± 0.24 mmol/L) [13]. Hypophosphataemia as an adverse event was reported in only 6 of 244 patients in that study, and none in a follow-up maintenance study of 104 patients [12], presumably due to most cases being asymptomatic.

Though most cases of hypophosphataemia appear transient, a minority of patients have persistent reduction in serum phosphate for up to several months. In our current study, serum phosphate remained lower at Day 28 compared to baseline (mean 0.95 vs 1.10 mmol/L, *p* = 0.001), and in 3 patients remained below 0.6 mmol/L. In another recent study, 56.9% of 52 patients receiving FCM were noted to have moderate to severe hypophosphataemia (defined in this study as < 0.65 mmol/L) at 2 weeks, with 13.7% of patients continuing to have serum phosphate below this level at 6 weeks, and some for up to 6 months [17]. Retrospective studies have also reported hypophosphatemia for as long as 6 months following intravenous iron [16, 23].

This persistent reduction in serum phosphate, particularly in the context of repeated iron infusions, may contribute to hypophosphatemic osteomalacia with fractures [18, 24, 25], which may have delayed clinical recognition due to the non-specific nature of symptoms reported by patients and often normal plain radiography. Given that patients with IBD may have nutritional deficiencies and reduced bone density, they may be particularly susceptible to these complications [19]. Furthermore, iFGF-23 is also known to be upregulated by systemic inflammation, potentially further predisposing to hypophosphataemia [20]. Nonetheless, baseline levels of iFGF-23 were similar in patients with and without IBD in the current study. Though not performed in this study, measurement of bone turnover markers following FCM in a future study may help to stratify risk of osteomalacia in the long-term.

Eliciting risk factors for hypophosphataemia following FCM, especially in patients with IBD is, therefore, crucial, to develop potential preventative strategies. The absence of any association between systemic or intestinal inflammation, or vitamin D components (25-hydroxy vitamin D, 1,25(di-OH) D, free or bioavailable 25-hydroxy vitamin D), with risk of hypophosphataemia, means that correction of these factors may not be the answer.

Interestingly, a significant correlation between DBP and the lowest serum phosphate was noted. DBP is a liver-derived α-globulin structurally similar to albumin, which binds about 85–90% of circulating vitamin D metabolites [26]. DBP may control the availability of vitamin D metabolites, especially 25-hydroxy vitamin D, to tissues by allowing only the small free fraction to passively enter cells through diffusion across cell membranes, or actively via interaction with membrane glycoproteins megalin and cubulin [27]. Higher concentrations of DBP may directly reduce circulating 1,25 dihydroxy vitamin D [28]. In contrast, FGF-23 inhibits cytochrome P27B1, the enzyme which 1-hydroxylates 25-hydroxy vitamin D to 1,25 dihydroxy vitamin D. The potential relationship between DBP, FGF-23, 1,25 dihydroxy vitamin D and serum phosphate warrants further study.

The mechanism of hypophosphataemia following intravenous iron administration, specifically after FCM, has been investigated in numerous studies [15, 29, 30]. Consistent with previous reports, the hormone iFGF-23, which primarily inhibits renal phosphate reabsorption producing phosphate wasting but also reduces circulating 1,25(di-OH) D and thus intestinal phosphate absorption [15, 29, 30], was demonstrated to have significantly risen by Day 2 in our study. Changes in C-terminal FGF-23 should generally be interpreted with some caution as the assay detects both cFGF-23 fragments and the intact molecule. Given that levels remained relatively stable, it is likely that impaired intracellular degradation of iFGF-23 is the likely explanation for the observed changes in FGF-23 [15]. The timing of peak of iFGF-23 is uncertain, with initial studies describing a peak at day 1 [15], and other studies reporting a peak at Day 2 [29, 30]. Both days 1 and 2 have not been published in a single study, and hence the precise trajectory of iFGF-23 in the first 2–3 days remains uncertain. The decline by day 7 has been consistently reported previously [15, 29, 30].

Though patients with and without IBD did not differ in rates of hypophosphataemia, patients with CD had a higher risk of hypophosphataemia than those with UC. Subgroup analysis based upon the location of CD was not possible, since only 2 of 11 patients with CD had isolated colonic disease, with most having ileal or ileocolonic disease. Given that phosphate is absorbed in the small intestine, the difference between patients with CD and UC might be accounted for by a greater susceptibility to malabsorption of phosphate in patients with CD and requires further investigation. It is worth noting, however, that baseline phosphate was similar in patients with CD and UC (*p* = 0.34).

FCM is one of the most commonly prescribed formulations of intravenous iron worldwide, but emerging studies demonstrate a significantly higher risk of hypophosphataemia following FCM compared to other intravenous iron formulations such as iron dextran, iron isomaltoside, and ferumoxytol [15, 17, 31]. The reasons for this difference are unclear, but may be secondary to a differential effect on cleavage of iFGF23 in osteocytes by differing carbohydrate moieties [15]. Although apparently consistent, such serological effects need to be balanced against the relative clinical safety and the limited sequelae of FCM noted to date, particularly in comparison to other formulations such as iron dextran. Longer studies with more rigorous endpoints will enable a clearer distinction to be made.

The strength of this study lies in the uniform, prospective collection of data in patients with and without IBD, and its ability to clarify rates of hypophosphataemia as well as pertinent risk factors. Nonetheless, it must be acknowledged that fractional urinary phosphate excretion was not measured in patients. Previous studies have shown that an increase in phosphaturia is the primary mechanism for hypophosphataemia following intravenous iron, but whether this effect differs between patients with and without IBD, or is influenced by systemic inflammation, is an area for further study. Secondly, the duration of persistence of hypophosphataemia beyond 4–6 weeks and effect on bone turnover markers in this population remains undetermined, particularly in relation to the presence or type of IBD, and remains an avenue for further investigation.

## Conclusions

Hypophosphataemia following FCM occurs at similar rates in patients with and without IBD, and is not influenced by inflammation, or vitamin D status. Alternative intravenous iron formulations associated with a lower risk of hypophosphataemia might be considered for iron replacement in such patients.

## Supplementary information

**Additional file 1:Table S1.** Delayed adverse events (from 1 h after infusion to 28 days follow-up). **Figure S1.** Correlation between minimum serum phosphate during follow-up and multiple markers in patients with IBD.

## Data Availability

The authors declare that the data generated or analysed in this study are available within the paper and its supplementary materials.

## Abbreviations

CD: Crohn’s disease
CTCAE: Common Terminology Criteria for Adverse Events
DBP: Vitamin D binding protein
ELISA: Enzyme-linked immunosorbent assay
FCM: Ferric carboxymaltose
FGF23: Fibroblast growth factor-23
cFGF: C-terminal fibroblast growth factor-23
iFGF: Intact fibroblast growth factor-23
IBD: Inflammatory bowel disease
UC: Ulcerative colitis
