# Supplementary material for: Hypophosphataemia after ferric carboxymaltose is unrelated to symptoms, intestinal inflammation or vitamin D status
Source: BMC Gastroenterol. 2020 Jun 10;20:183. doi: 10.1186/s12876-020-01298-9 (PMC7288415; doi:10.1186/s12876-020-01298-9)
Supplement: Supplementary file 1 — Additional file 1:Table S1. Delayed adverse events (from 1 h after infusion to 28 days follow-up). Figure S1. Correlation between minimum serum phosphate during follow-up and multiple markers in patients with IBD. [file 12876_2020_1298_MOESM1_ESM.docx]

**Supplementary Table 1.** Delayed adverse events (from 1 hour after infusion to 28 days follow-up).

| Adverse event | IBD (n, %) | Non-IBD (n, %) |
| --- | --- | --- |
| Headache^†^ | 0 (0) | 3 (15) |
| Arthralgia / myalgia^†^ | 6 (25) | 5 (25) |
| Fatigue^†^ | 3 (13) | 3 (15) |
| Nausea / vomiting | 1 (4) | 3 (15) |
| Abdominal pain | 2 (8) | 2 (10) |
| Diarrhoea | 1 (4) | 1 (5) |
| PR bleeding | 0 (0) | 1 (5) |
| Dyspnoea^†^ | 0 (0) | 1 (5) |
| Dizziness^†^ | 0 (0) | 1 (5) |
| Palpitations | 0 (0) | 1 (5) |
| Rash | 1 (4) | 0 (0) |

^†^ symptoms considered as possibly or likely due to ferric carboxymaltose

**Supplementary Figure 1.** Correlation between minimum serum phosphate during follow-up and multiple markers in patients with IBD

| A  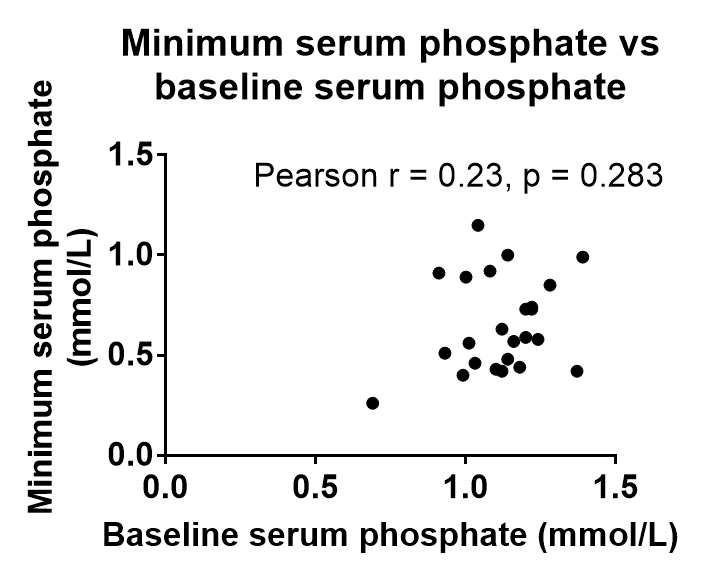 | B  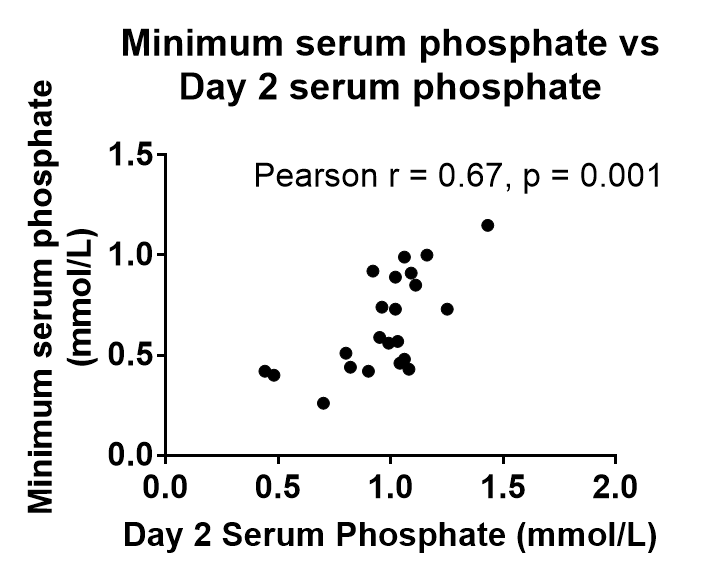 |
| --- | --- |
| C  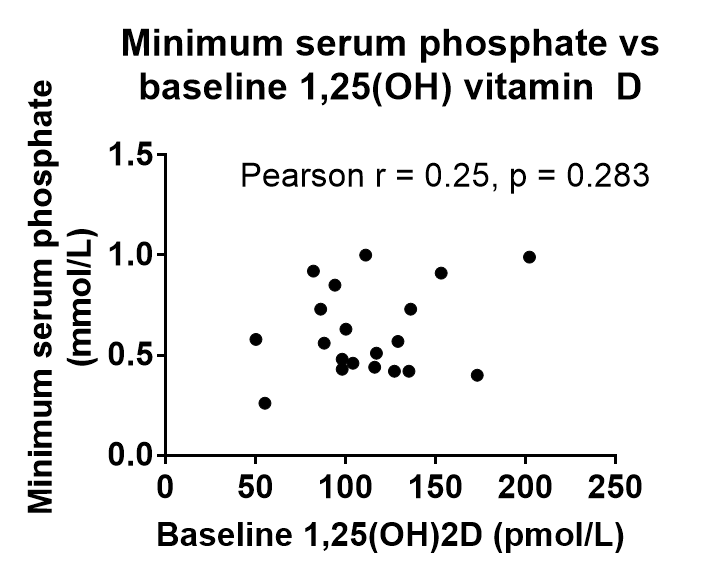 | D  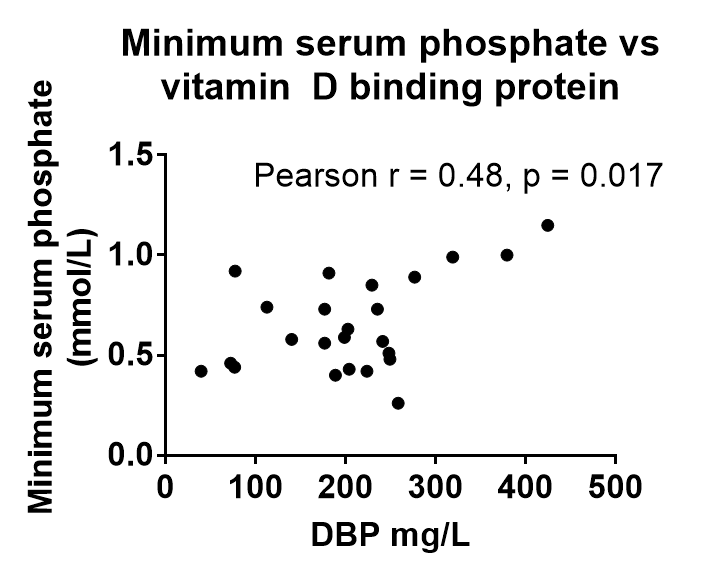 |
| E  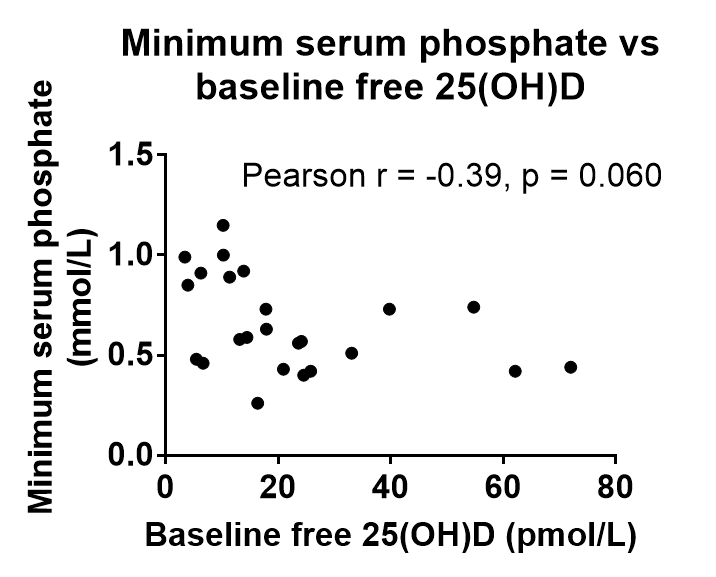 | F  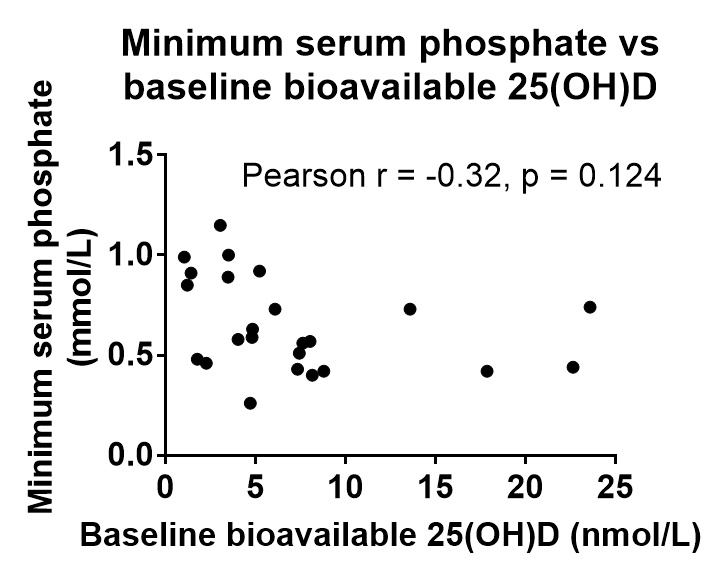 |
